# Use of trimethoprim- sulfamethoxazole for treating *Pneumocystis jirovecii pneumonia* in a patient with glucose-6-phosphate dehydrogenase deficiency: a case report

**DOI:** 10.3389/fmed.2024.1443645

**Published:** 2024-09-10

**Authors:** Linyu Wang, Xianlong Xie, Zhe Li, Yan Li

**Affiliations:** ^1^Department of Pharmacy, Guangxi Medical University Cancer Hospital, Nanning, China; ^2^Department of Intensive Care Unit, Guangxi Medical University Cancer Hospital, Nanning, China; ^3^Department of Haematology/Oncology and Paediatric Oncology, Guangxi Medical University Cancer Hospital, Nanning, China

**Keywords:** trimethoprim-sulfamethoxazole, *Pneumocystis jirovecii* pneumonia, glucose-6-phosphate dehydrogenase, lymphoma, hemolysis, case report

## Abstract

**Background:**

*Pneumocystis jirovecii* pneumonia (PJP) is an opportunistic infection caused by the yeast-like fungus *P. jirovecii*. As recommended by some guidelines, the first-line treatment for this infection is trimethoprim-sulfamethoxazole (TMP-SMX), and the second-line treatment includes drugs such as dapsone, pentamidine, primaquine, Atovaquone, clindamycin, and caspofungin. Glucose-6-phosphate dehydrogenase (G6PD) deficiency is an X-linked gene disorder in which treatment with oxidizing drugs, such as sulfonamides, dapsone, primaquine, can directly destroy hemoglobin present in red blood cells (RBCs), thereby inducing methemoglobin and hemolysis.

**Case presentation:**

Here, we present the case of a lymphoma patient with previous G6PD deficiency who was admitted to ICU for the treatment of severe pneumonia combined with respiratory failure. PJP was detected by the next-generation sequencing of the bronchoalveolar lavage fluid. The patient was initially treated with the antifungal drug caspofungin; however, this treatment showed poor therapeutic effect. Based on the evaluation of G6PD enzyme activity and the patient’s previous history of G6PD deficiency, we finally treated the patient with low-dose TMP-SMX combined with caspofungin and provided rigorous medical care to the patient. Following this treatment, the patient’s clinical symptoms improved, lung computed tomography showed reduced pulmonary inflammation, and the fungal β-(1,3)-D-glucan test (G test) showed decreased levels of fungal D-glucan. After 57 days, the TMP-SMX treatment was discontinued. No symptoms related to G6PD deficiency, such as hemolysis, hematuria, and anemia, occurred during the treatment course.

**Conclusion:**

This is the first report mentioning the successful treatment of *Pneumocystis jirovecii* pneumonia with a double-drug regimen with low-dose TMP-SMX and caspofungin in a T-lymphoblastic leukemia/lymphoma patient with previous G6PD deficiency. Enzyme activity detection is the first step for anti-PJP treatment in patients with G6PD deficiency. Although patients with mild enzyme deficiency may not show any adverse reactions, we still recommend the regular monitoring of the levels of RBCs, hemoglobin, and hematocrit before and after the use of sulfonamides or sulfoxides and other oxidizing drugs in patients with G6PD deficiency. Among other things, early and correct diagnosis of *Pneumocystis jirovecii* pneumonia in hematological malignancies patients is very important. Relevant oncologists should be alert to the risk of *Pneumocystis jirovecii* pneumonia in these patients.

## Introduction

*Pneumocystis jirovecii* pneumonia (PJP) is a fungal infection caused by *P. jirovecii*. Based on the published guideline (1), the first-line treatment for PJP in patients with hematological malignancies, solid organ transplants, cancers, and autoimmune diseases is trimethoprim-sulfamethoxazole (TMP-SMX) at the doses of 15–20 mg/kg (TMP) and 75–100 mg/kg (SMX) for ≥14 days. The second-line treatment includes drugs such as dapsone, pentamidine, primaquine, Avatorone, and clindamycin. Additionally, because of the presence of (1,3)-β-D-glucan in the cell wall of *P. jirovecii*, echinocandins (such as caspofungin and micafungin) can kill the cells of *P. jirovecii* by inhibiting the synthesis of (1,3)-β-D-glucan (2). The guidelines recommend the combination of caspofungin and TMP-SMX as the second-line treatment for anti-PJP therapy (1). In China, based on the «Chinese expert consensus on diagnosis and treatment of pneumocystis pneumonia in AIDS patients (2024 edition)» (3), the first-line drug for treating PJP is TMP-SMX; its dose is adjusted according to patient tolerance, and the course of treatment is extended appropriately according to efficacy and adverse reactions. The alternative drugs are a combination of dapsone, clindamycin, primaquine, and caspofungin.

Glucose-6-phosphate dehydrogenase (G6PD) deficiency is caused by mutations in the *G6PD* gene, resulting in varying degrees of enzyme deficiency and changes in protein expression, leading to various clinical subtypes. G6PD deficiency induces red cells to become highly vulnerable to oxidative damage and consequently susceptible to hemolysis. The prevalence of G6PD deficiency in the overall population of China is 2.1% (4), and to date, over 35 different G6PD gene mutations have been reported, with G6PD Kaiping and G6PD Canton predominating in earlier investigations (5). Patients with G6PD deficiency usually do not show apparent clinical manifestations, and the disease is diagnosed only if the patients receive any exogenous stimulation such as administration of antimalarial drugs (primaquine, chloroquine, quinine, pentaquine, and adipine), sulfones (thiazole sulfone and aminophene sulfone), sulfonamides (sulfamethoxazole, sulfadimethoxine, sulfapyridine, and salazosulfapyridine), antipyretics (acetazolamide and acetanilide), chloramphenicol, and isoniazid. According to previous reports, patients with G6PD deficiency are prohibited from using the following seven drugs: dapsone, methylthioninium chloride (methylene blue), nitrofurantoin, phenazopyridine, primaquine, rasburicase, and tolonium chloride (6).

Here, we present the case of a lymphoma patient with previous G6PD deficiency who was initially treated with the antifungal drug caspofungin and later with low-dose (5 mg/kg/d TMP) TMP-SMX combined with caspofungin. Following this treatment, the patient’s clinical symptoms improved, lung computed tomography showed reduced pulmonary inflammation, no hemolysis or cytopenia occurred following treatment with TMP-SMX tablets.

## Case presentation

A 33-year-old man was diagnosed to have lymph node T-LBL/acute T-lymphoblastic leukemia in February 2023. VDLD (vincristine, daunorubicin, L-asparaginase, dexamethasone) induction chemotherapy was started on May 15, 2023, with the following specific doses: vincristine 2 mg (d1, 8, 15, and 22) IV, daunorubicin 50 mg (d1, 8, 15, and 22) IV, peraspartase 3750 IU (d9 and 23) IV, and dexamethasone 15 mg (d122) IV. On June 25, 2023, the patient developed cough, mainly dry cough, and phlegm, and he visited the local hospital for treatment. He was diagnosed to have pneumonia and was treated for infection, rehydration, and fever; however, the therapeutic effect was poor, and the patient later developed respiratory failure. On July 1, 2023, he was transferred to the ICU of our hospital. He was diagnosed to have severe pneumonia and was treated with tracheal intubation and ventilator-assisted breathing, anti-infection medication, vasoactive drugs, fluid replacement to maintain blood pressure, relieve respiratory spasmand and nutritional support. The patient had previous history of G6DP deficiency.

**FIGURE 1:**
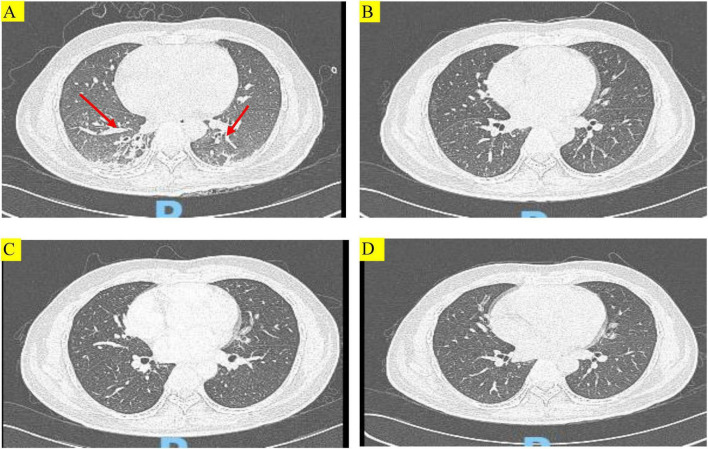
Chest computed tomography (CT) images of the patient during treatment. **(A)** Chest CT showing multiple patchy and striped shadows (red arrows) before treatment with TMP-SMX. **(B–D)** Chest CT scans conducted at 14 days after treatment with TMP-SMX, 29 days after discharge, and 57 days after discharge, respectively. Multiple patchy and striped shadows were not visible in both lungs after treatment.

The patient’s physical parameters were as follows: height: 165 cm, weight: 60 kg, body temperature: 38.1°C, pulse rate: 89 times/min, respiration rate: 40 times/min, blood pressure: 115/78 mm Hg (noradrenaline maintenance). Laboratory results revealed type I respiratory failure, leukocytosis and hypoproteinemia, severe anemia, elevated C-reaction protein and procalcitonin level, type I respiratory failure. The renal profiles were within normal limits. Sputum cultures did not isolate any respiratory pathogens, APACHE-II score was high (Table 1). The chest CT on admission exhibited patchy shadows in both lung fields, and revealed bilateral pneumonia and bilateral pleural effusion (Figure 1A).

**TABLE 1:** Laboratory test on admission.

Laboratory parameters	Value	Units	Reference ranges
pH	7.45		7.35–7.45
Oxygen concentration	45.00	%	21–100
Partial pressure of carbon dioxide	43.7	mm Hg	35–45
Partial pressure of oxygen	73.5	mm Hg	83–108
Oxygenation index temperature correction	163	%	400–500
White blood cells	23.55	10^9^/L	3.5–9.5
Neutrophils%	90.00	%	40–75
Red blood cells	1.94	10^12^/L	3.8–5.1
Hemoglobin	56	g/L	120–160
C-reactive protein(CRP)	113.90	mg/L	0–10
Procalcitonin(PCT)	1.97	ng/mL	<0.05
Albumin	21.5	g/L	35–55
Prealbumin	177	mg/L	200–400
Creatinine	72	μmol/L	50–110
Lactate dehydrogenase(LDH)	830	U/L	103–227
APACHE-II score	14		

Meanwhile, the patient experienced daily fever up to 38.1°C, and had a dry cough with phlegm. He was thus empirically treated with cefoperazone, the initial anti-infection regimen was imipenem-cilastatin + vancomycin + caspofungin. At the time of admission, the patient was diagnosed to have (1) severe pneumonia; (2) acute respiratory failure (type I respiratory failure); (3) T-lymphoblastic leukemia/lymphoma (stage IV group A); (4) G6PD deficiency-associated anemia.

## Therapeutic process

The patient was admitted to our hospital and administered an initial empirical treatment with imipenem-cilastatin + vancomycin + caspofungin. On July 03, *Pneumocystis jirovecii* was detected in the next-generation sequencing of the patient’s bronchoalveolar lavage fluid sample. The diagnosis of PJP depends on the detection of cysts or trophoblasts in microscopy examination. Because of the low load of *P. jirovecii* in most non-HIV patients, the sensitivity of traditional smear microscopy is low; consequently, the early diagnosis of non-HIV PJP is difficult and could be easily missed or delayed. The mNGS is superior to several common clinical methods for diagnosing suspected PJP. The serum G test together with mNGS can further improve the diagnostic efficacy of mNGS (7). The 2016 European Conference on Leukemia Infections (ECIL) recommended early initiation of anti-pneumocystis treatment for highly suspected patients, independent of bronchoscopy results. In the present case, fever, dry cough, and phlegm were the main symptoms. Based on the patient’s clinical symptoms, increase in the G test, and lung CT findings, the patient was clinically diagnosed to have *P. jirovecii* pneumonia (PJP). However, considering that the patient had lymphoma and G6PD deficiency, we were concerned that the use of sulfonamides would cause intractable hemolysis. Moreover, there is a case report of an HIV-infected patient, diagnosed as PJP with G6PD deficiency, who was successfully treated with anidulafungin for 3 weeks with successful results (8). Caspofungin, like anidulafungin, is one of the echinocandins, which can kill pneumocystis by inhibiting the synthesis of (1,3)-β-D-glucan. Hence, we continued treatment with the anti-PJP agent caspofungin. On July 11, after 12 days of treatment with caspofungin, the G test remained high, and a repeated lung CT scan revealed no resolution of pulmonary infection. Considering the poor anti-PJP effect of caspofungin, the young age of the patient, and the subsequent delay in follow-up lymphoma chemotherapy in the absence of an appropriate anti-PJP treatment, we decided to change the anti-PJP treatment regimen.

Based on the WHO guidelines (9), G6PD enzyme deficiency is classified into 5 categories according to the enzyme activity level, and categories IV and V are not considered clinically significant. Oxidizing drugs can be administered in standard doses to these patients, provided that hemolytic reaction is strictly monitored (2). Therefore, in the present case, we conducted G6PD enzyme deficiency screening by a colorimetric method. The G6PD enzyme activity level of the patient was 0.65 (normal value: 1.00–2.30). This result implied that the G6PD enzyme activity level of the patient was 65% of the normal value. Thus, the patient was considered to have grade IV G6PD enzyme deficiency and therefore could be treated with oxidizing drugs at standard doses. After asking the patient that he had no previous history of G6PD deficiency, we developed two treatment protocols based on the severity of G6PD deficiency of the patient and drug availability:

**Protocol 1:** Low-dose TMP-SMX (TMP 5 mg/kg/d) (10–12) combined with caspofungin: TMP-SMX 2 tablets (160 mg TMP and 800 mg SMX) q12h po (starting with 1 tablet, administered daily until maintenance therapy) + caspofungin 50 mg qd ivgtt.
**Protocol 2**: Clindamycin 0.6 g q8h ivgtt + caspofungin 50 mg qd ivgtt (13, 14).

In order to achieve rapid control of the disease and improve the prognosis, we chose Protocol 1 as the treatment regimen. The following medication protocol was formulated by clinical pharmacists: (1) After 2 days of maintenance treatment, blood samples were analyzed to estimate the peak concentration of SMX (2–3 h after oral administration), and the recommended range was 100–200 mg/L (1); (2) The hemoglobin level, RBC count, urine color, presence of occult blood in urine, and skin/sclera color were monitored, and the treatment was discontinued if a decrease in RBC count or hemolysis was observed; (3) Following 21 days of treatment, the CD4^+^ T cell and lymphocyte counts were reviewed monthly. If CD4^+^ T cell count was <200/μL (1) or the lymphocyte count was <0.5 × 10^9^/L (15), TMP-SMX tablet was given at the dose of 1 tablet (80 mg TMP and 400 mg SMX) qd po to control the infection; and (4) The G test and CT scans were conducted to evaluate the therapeutic effect.

**FIGURE 2:**
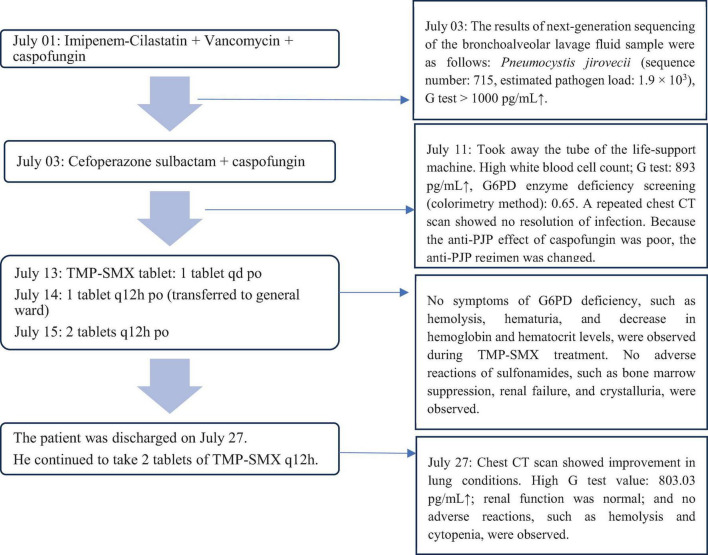
Changes in the patient’s condition at admission and after anti-PJP treatment.

No hemolysis or cytopenia occurred following treatment with TMP-SMX tablets, and the maintenance therapy was initiated on July 15. On July 27, after 14 days of treatment, the patient’s condition improved, and a repeat chest CT showed that multiple patchy shadows and stripe shadows in both lungs were obviously absorbed (Figure 1B). Subsequently, the patient was discharged from the hospital. Although the G test was still high (564.2 pg/mL), the patient was discharged and advised to undergo further treatment with TMP-SMX tablets by 2 tablets (160 mg TMP and 800 mg SMX) q12h. Figure 2 shows the treatment process.

## Follow-up and prognosis

On August 21, the patient was admitted to our hospital for chemotherapy. He had no cough, phlegm, and shortness of breath. A CT scan showed pulmonary inflammation (Figure 1C), and the G test was 242.17 pg/mL↑. The patient was recommended to continue with oral TMP-SMX tablets by 2 tablets (160 mg TMP and 800 mg SMX) q12h.

The patient was again admitted to our hospital on September 7 for re-examination. The spot shadow and the cord-like shadow observed in the lower lung in the lung CT scan were approximately of the same size as those noted before (Figure 1D). Following the treatment regimen for pneumonia, the G test was 76.11 pg/mL. Once PJP was controlled, the patient was asked to discontinue TMP-SMX tablets. No hemolytic reaction occurred during the examination period.

In the recent follow-up examination, the patient was regularly receiving chemotherapy with cyclophosphamide + cytarabine + mercaptopurine, and no cough, sputum, or shortness of breath was observed. Figure 1 shows changes in the findings of chest CT scans.

## Discussion

Presently, the diagnosis of G6PD deficiency primarily involves the enzyme activity detection assay, based on the range of enzyme activity in genotypes and heterozygotes. The WHO has classified G6PD deficiency into five grades: grade I indicates severe deficiency with chronic nonspherical hemolytic anemia, grade II also indicates severe deficiency (enzyme activity range: 1–10%), grade III represents moderate deficiency (enzyme activity range: 10–60%), grade IV implies normal deficiency (enzyme activity range: 60–150%), and grade V indicates increased enzyme activity (>150%) (9). Only grades I, II, and III G6PD deficiency are clinically significant, while grades IV and V G6PD deficiency can be treated with standard doses of oxidizing drugs (2).

In addition to TMP-SMX, other treatments are available for G6PD-deficient individuals with PJP. Nonoxidizing drugs such as Atovaquone, pentamidine, clindamycin, and caspofungin have also been used as second-line treatments for patients with PJP (16).

1) Atovaquone can be used alone and are relatively safe for patients with G6PD deficiency; For patients with G6PD deficiency, we recommend that Atovaquone 750 mg be taken twice a day for oral treatment or 1500 mg once daily for prevention (17). However, this drug is not marketed in China and are not registered with the National Medical Products Administration in China, so we could not obtain them for this patient.
2) Pentamidine is a recommended alternative drug for PJP in the current guidelines, including 3∼4 mg/kg, given intravenously once a day, or 300 mg monthly atomization therapy to prevent PJP (17). The efficacy is worse than that of Atovaquone, TMP-SMX, or dapsone, but its occurrence of hemolytic anemia in patients with G6PD deficiency is minor. So, it is also our recommended drug. The same as Atovaquone, pentamidine is not marketed in China and is not registered with the National Medical Products Administration in China.
3) Clindamycin is a lincomycin derivative and has good antimicrobial activity against Gram-positive bacteria and Gram-negative anaerobes. However, the precise mechanism through which it can treat PJP remains unclear (18). As an oxidant, primaquine has a particular effect on G6PD deficiency patients, while the combination of clindamycin with low-dose primaquine has a lower risk of hemolytic anemia for patients with G6PD deficiency (13, 19–22); moreover, clindamycin and caspofungin combination therapy for patients with G6PD deficiency has also been reported (13), which might be beneficial for patients with PJP who have failed treatment or are intolerant to TMP-SMX. Caspofungin, clindamycin, and intravenous TMP-SMX have been successfully used to treat moderate-to-severe PJP in immunocompromised patients (14).
4) Caspofungin mainly act on cysts and damage the structural and functional integrity of the cell wall by inhibiting β-D-glucan synthesis in the cyst wall of *Pneumocystis jirovecii*, and these drugs do not induce hemolysis. The ECIL guideline and Chinese expert consensus (1, 3) have suggested that caspofungin is effective as a salvage treatment. Because the cyst-to-trophoblast ratio is ≈1:9 during pulmonary infection (23), caspofungin alone are not recommended for treating PJP infection, particularly for patients with severe PJP or refractory PJP, and these drugs should be combined with TMP-SMX for a synergistic effect (24). Currently, there are no large-scale prospective studies on using caspofungin to treat PJP, and many case reports or cohort studies have reported the clinical data of using caspofungin as a remedial measure after the failure of TMP-SMX (25–27). Similarly, in a European retrospective clinical study of HIV patients with PJP, 10 patients could not tolerate the first-line therapy and were treated with caspofungin. Of these 10 patients, 8 patients were successfully treated (one patient died due to pneumothorax, and one patient died due to lymphoma) (27).

To avoid hemolytic reaction as much as possible, our Protocol 2 did not use dapsone, primaquine, and other oxidizing drugs; instead, we chose the relatively safe combination of clindamycin and caspofungin. Although this strategy seems aggressive, TMP-SMX combined with caspofungin was selected to ensure the best curative effect.

The standard dose for PJP treatment is 15–20 mg/kg (TMP) and 75–100 mg/kg (SMX) per day. A treatment duration of ≥14 days is preferred. Considering that the patient had lymphoma and was admitted to hospital with the suspicion of G6PD deficiency-related anemia, although the hemoglobin level was restored to the normal level after treatment, the patient was worried about the occurrence of hemolysis after TMP-SMX treatment. The patient was more likely to suffer from hemolysis if he received the conventional administration dose (15–20 mg/kg/d TMP). Low doses of TMP-SMX (<15 mg/kg/d TMP) significantly reduced adverse reactions and had similar survival rates. Hence, a low-dose TMP-SMX regimen should be considered a viable option for treating PJP, as it may reduce treatment discontinuation rates and reduce patient harm (10). Therefore, we administered TMP-SMX at a low dose (5 mg/kg/d TMP), which led to a longer treatment course due to a low drug concentration.

Patients with stage IV group A lymphoma may show a decreased RBC count following disease progression; this manifestation might be confused with symptoms associated with G6PD enzyme deficiency. In this clinical scenario, regardless of whether the decreased RBC count is due to the hemolytic reaction induced by sulfonamide use, the drug administration should be immediately stopped, and the current anti-PJP treatment regimen should be changed.

Based on the treatment process used for our patient, we summarize the appropriate treatment approach for similar patients as follows. For patients diagnosed to have G6PD deficiency, the first step is to detect the enzyme activity of G6PD, select a suitable medication regimen according to the enzyme activity level, and choose non-oxidizing drugs with accurate efficacy as far as possible. If sulfonamides and primaquine are chosen, a low dose should be administered initially, and the dose should be increased stepwise to achieve the maintenance dose. Furthermore, patients with grade IV G6PD enzyme deficiency should also be closely monitored for hemolysis-related event indicators and clinical manifestations such as cytopenia, anemia, decreased hematocrit levels, and darker urine color, and drug withdrawal and blood transfusion should be promptly implemented, if necessary. The adverse reactions of TMP-SMX are renal injury, crystalluria, and bone marrow suppression, which are related to the drug concentration. Therefore, it is recommended to monitor the blood peak concentration of SMX, if such conditions exist. In the present case, PJP treatment was evaluated according to the clinical symptoms, chest CT scan findings, and the fungal D-glucan level in the G test, and the duration of TMP-SMX administration was accordingly determined.

In HIV patients, the count of CD4^+^ T cells is a helpful marker to classify the risk of PJP. PJP patients require primary preventive treatment at the CD4^+^ cell count of <200/mm^3^. However, in patients without AIDS, there are no clinical guidelines established for PJP prophylaxis, particularly for patients with autoimmune diseases treated with glucocorticoids, cytotoxic drugs, or biological agents. A previous study identified a total lymphocyte count of <0.5 × 10^9^/L as an independent risk factor for developing PJP (15). Other authors have also recommended PJP prophylaxis in patients with a total lymphocyte count of <0.8 × 10^9^/L (28). In these cases, the counts of CD4^+^ T cells and lymphocytes were reviewed monthly after drug withdrawal to determine whether daily medication was required to prevent PJP recurrence.

## Conclusion

We used double combination therapy with low-dose TMP-SMX and caspofungin to treat PJP in a T-lymphoblastic leukemia/lymphoma patient with previous G6PD deficiency. Given the high mortality in PJP in HIV-uninfected hematological malignancies patients, we adopted an aggressive anti-PJP regimen. With this double drug regimen, our patient’s respiratory condition was apparently improved, and a repeat chest CT showed that multiple patchy and striped shadows in both lungs were obviously absorbed, and the patient was successfully discharged from the hospital, no hemolytic reaction occurred during the examination period. Importantly, the patient was regularly receiving chemotherapy with cyclophosphamide + cytarabine + mercaptopurine, and no cough, sputum, or shortness of breath was observed. are at high-risk of developing PJP and this diagnosis should be considered early in patients with clinical and radiological features suggestive of PJP.

Enzyme activity detection is the first step for anti-PJP treatment in patients with G6PD deficiency. The extent of G6PD deficiency varies according to the enzyme activity. Although patients with mild enzyme deficiency may not show any adverse reactions, we still recommend the regular monitoring of the levels of RBCs, hemoglobin, and hematocrit before and after the use of sulfonamides or sulfoxides and other oxidizing drugs in patients with G6PD deficiency. Long-term follow-up of patients should also be conducted, and patients and their families should be advised regarding prohibited foods and careful use of drugs to prevent hemolytic diseases. Among other things, early and correct diagnosis of PJP in hematological malignancies patients is very important. Relevant oncologists should be alert to the risk of PJP in these patients.

Our study had a limitation. During the treatment, for the reason of lacking instruments, we did not monitor the peak concentration of SMX, so the patient used TPM-SMX 57 days, exceeding the recommended course of treatment, and we did not know whether it was because the effective blood concentration was not reached, or the TPM-SMX resistance was developed.

## Data Availability

The raw data supporting the conclusions of this article will be made available by the authors, without undue reservation.
