# Supplementary material for: Use of trimethoprim- sulfamethoxazole for treating Pneumocystis jirovecii pneumonia in a patient with glucose-6-phosphate dehydrogenase deficiency: a case report
Source: Front Med (Lausanne). 2024 Sep 10;11:1443645. doi: 10.3389/fmed.2024.1443645 (PMC11420125; doi:10.3389/fmed.2024.1443645)
Supplement: Supplementary file 1 [file Data_Sheet_1.docx]

Supplementary Material

Use of Trimethoprim-sulfamethoxazole for treating *Pneumocystis* *jirovecii pneumonia* in a patient with glucose-6-phosphate dehydrogenase deficiency: A case report

# Linyu Wang1 †, Xianlong Xie2 †, Zhe Li3, Yan Li1*

# 1 Department of Pharmacy, Guangxi Medical University Cancer Hospital, Nanning, China.

# 2 Department of Intensive Care Unit, Guangxi Medical University Cancer Hospital, Nanning, China

# 3 Department of Haematology/Oncology and Paediatric Oncology, Guangxi Medical University Cancer Hospital, Nanning, China

# * Correspondence:

# Yan Li

# [liyan_gx@126.com](mailto:liyan_gx@126.com)

†These authors contributed equally to this work.

# Supplementary Figures and Tables

For more information on Supplementary Material and for details on the different file types accepted, please see [here](https://www.frontiersin.org/guidelines/author-guidelines#supplementary-material).

## Supplementary Figures

**Supplementary Figure 1.** **The trend of WBC and G test values.** From July 1 to July 12, the anti-PJP regimen was caspofungin, with a decrease in WBC, but the decrease in G test was not obvious. The TMP-SMZ tablet was started on July 13, and the maintenance dose was used on July 16. The G test was significantly decreased compared with that before when the patient was hospitalized for reexamination on August 21. On September 7, she was admitted to hospital for re-examination, and the WBC were normal, and the G test value dropped to nearly normal.

**Supplementary Figure 2.** **The trend of HB, HCT% and RBC values.** Since taking TMP-SMZ tablets on July 13, the three test indicators did not decreased, and no blood products or blood cell drugs were injected during the treatment.

**Supplementary Figure 3.** **The trend of lymphocyte values.** The lymphocyte count was normal during the treatment.


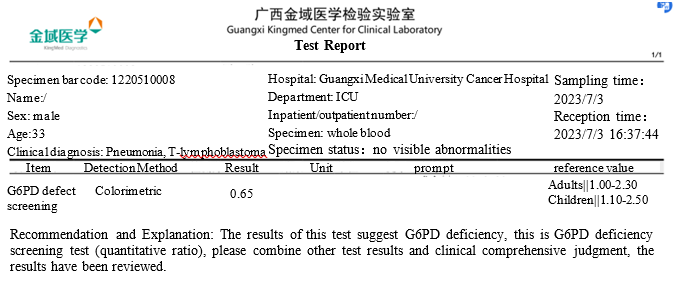


**Supplementary Figure 4.** The results of G6PD examination report


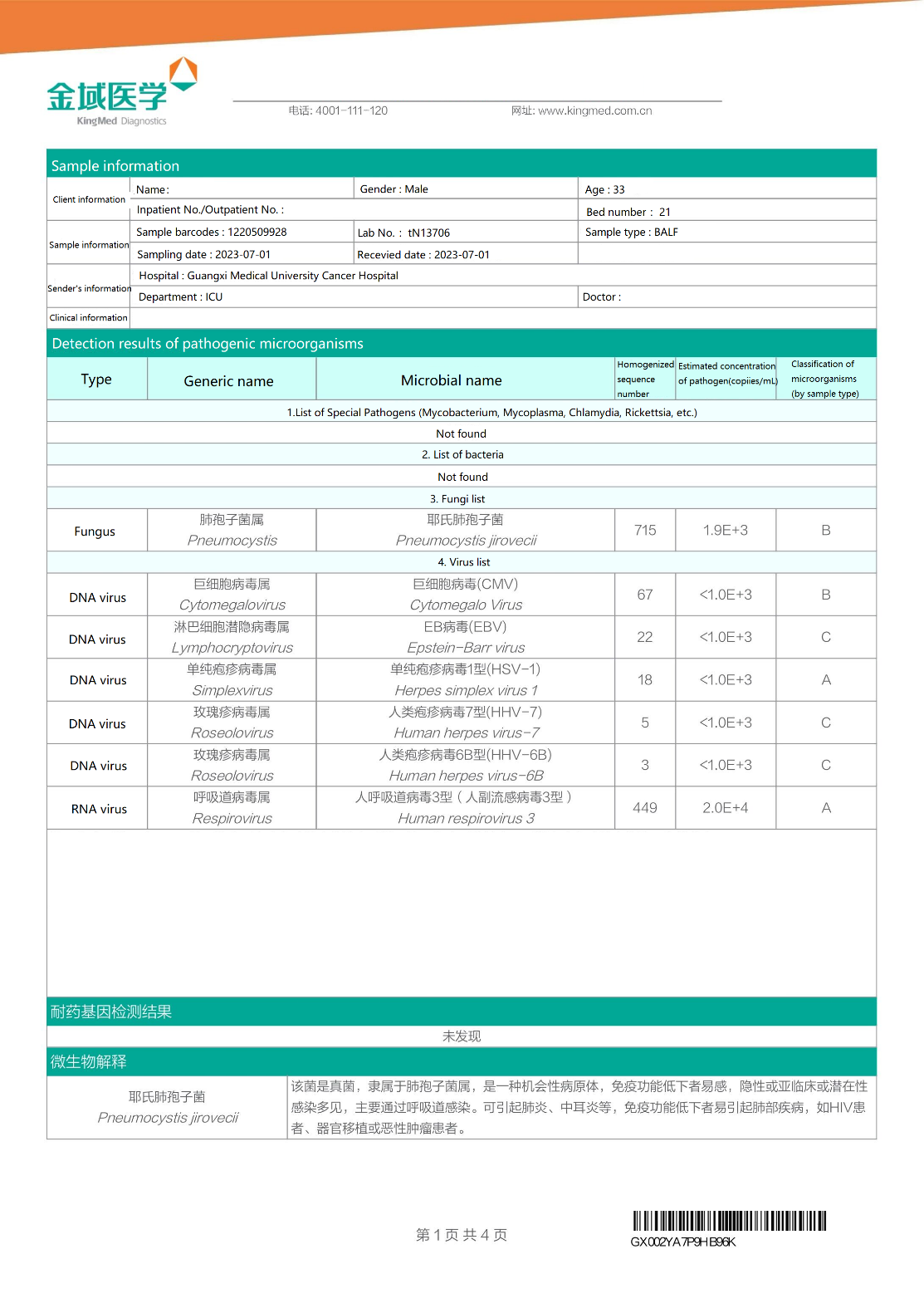


**Next-Generation Sequencing Report**

Notes：

Homogenized sequence number: Every 100K original sequence contains the sequence number of the microorganism. The higher the homogenized sequence number, the higher the reliability of the sample containing the microorganism.

Estimated pathogen concentrations (copies/mL) : The microbial content in samples is calculated using bioinformatic methods. This result is not an absolute quantity and is for clinical reference only.

Microorganism classification (by specimen type) Description:

A: It is an obligate pathogenic pathogen in respiratory tract specimens, or a common clinical pathogenic pathogen.

B: It is an opportunistic (conditional) pathogenic agent in respiratory tract specimens, and may be present when the patient has systemic or local immunosuppression/impairment/deficiency, respiratory barrier dysfunction, or lower respiratory microecological imbalance

If it causes infection, consider whether it is a pathogenic agent based on the actual clinical situation of the patient.

C: It is a normal microecological flora of the lower respiratory tract, which generally does not lead to infection, but there is the possibility of attracting lung abscess by mistake.

The above classification of pathogens is for clinical reference only, and the final interpretation of pathogens is subject to clinical practice, and the final interpretation is owned by clinicians.

**Supplementary Figure 5.** **The results of** **next-generation sequencing of the bronchoalveolar lavage fluid sample**
